# Synovial tissue features associated with poor prognosis in inflammatory arthritis

**DOI:** 10.1186/s13075-023-03255-9

**Published:** 2024-01-10

**Authors:** Ana Belén Azuaga, Andrea Cuervo, Raquel Celis, Beatriz Frade-Sosa, Juan C. Sarmiento-Monroy, Virginia Ruiz-Esquide, José A. Gómez-Puerta, Raimon Sanmartí, Julio Ramírez

**Affiliations:** 1 Rheumatology Department, Hospital Clinic of Barcelona, Barcelona, Spain; 2 Rheumatology Department, Hospital General de Granollers, Granollers, Spain

**Keywords:** Synovial tissue, Poor prognosis factor, Rheumatoid arthritis, Psoriatic arthritis, Undifferentiated arthritis

## Abstract

**Background:**

Inflammatory arthritis encompasses a group of immune-mediated diseases characterized by chronic joint inflammation. Despite having pathogenic mechanisms in common, the prognosis of rheumatoid arthritis (RA), psoriatic arthritis (PsA), and undifferentiated arthritis (UA) could be different regarding progression to chronic, to erosive, or to self-limited disease. Our aim was to evaluate the potential association of synovial tissue (ST) inflammatory cell infiltrate, the presence of ectopic lymphoid neogenesis (LN +) structures, and poor prognosis factors (PPF) in patients with RA, PsA, and UA.

**Methods:**

We conducted a retrospective study including patients with active arthritis (RA, PsA, UA) who had ST obtained by rheumatological arthroscopy or ultrasound-guided biopsy. Clinical, demographic, and immunohistochemical data of the synovium was evaluated. Patients with biological therapy at the time of synovial biopsy were excluded. PPF in patients with RA and UA were defined by the presence of anti-cyclic citrullinated peptide antibodies and/or rheumatoid factor, development of bone erosions, or requirement of biological therapy during the follow-up. PPF in patients with PsA were defined as the presence of high levels of acute-phase reactants (ESR/CRP), dactylitis or nail involvement at the time of biopsy, development of bone erosion, or requirement of biological therapy during the follow-up.

**Results:**

A total of 88 patients were included: 26 RA, 33 PsA, and 29 UA. All patients were followed up for 5 years after the biopsy. Fourteen (53.84%) RA patients had PPF, and 17 (65.38%) had LN + . LN + was associated with PPF (*p* 0.038) and biologic therapy initiation (*p* 0.018). A total of 14 (43.75%) PsA patients had PPF. CD15 infiltrate (410.68 [*SD* 477.63] cells/mm^2^) was associated with PPF (*p* 0.008) in PsA patients. Sixteen (55.17%) patients with UA had PPF, and 13 (44.82%) had LN + . In this group, synovial CD68 + macrophages cells density was negatively correlated with DAS28-CRP (*r* =  − 0.346, *p* 0.042).

**Conclusions:**

The presence of LN + and higher CD15 + polymorphonuclear cells infiltrate was associated with PPF in RA and PsA, respectively. No associations were found for UA. These findings suggest a great heterogeneity of the ST features and its pathogenic implications in the subtypes of inflammatory arthritis.

## Introduction

Rheumatoid arthritis (RA), psoriatic arthritis (PsA), and undifferentiated arthritis (UA) are chronic immune-mediated inflammatory diseases characterized by joint inflammation. Patients with UA can have different clinical presentations, such as monoarthritis of small or large joints, oligoarthritis, or polyarthritis. Recently, in a cohort of 310 UA patients, up to 14% developed RA within the first year of follow-up [1]. Clinical presentation, pathophysiology, and histopathological changes in ST may differ among the different types of inflammatory arthritis. Histology analysis of synovial tissue (ST) can aid in diagnosing and predicting outcomes. Previous research has examined the association between ST ectopic lymphoid neogenesis (LN +) structures and prognosis in RA and PsA [2–4].

LN are radial aggregates of T and B lymphocytes with dendritic cells characterized by the expression of peripheral node addressin positive (PNAd +) high endothelial venules (HEV) around blood vessels. These structures also express several chemokines involved in the traffic and tissue compartmentalization of T and B cells, resembling secondary lymphoid organs [2, 5].

In previous research in RA, LN + was found in 49% of the patients. This patients with ST LN + had a longer disease duration, higher prevalence of previous TNF inhibitors (TNFi), and less response to the therapies. Furthermore, in the subgroup of patients who underwent a second biopsy after treatment with TNFi, reversal of LN features occurred in 9 (56%) out of 16 patients who were LN + before treatment. The posttreatment disappearance of LN correlated with good clinical responses [3]. However, other groups reported opposite results [6, 7].

 ST LN + was identified in 13 out of 27 patients with PsA in another study. Interestingly, a subset of these patients exhibited LN regression following remission after TNFi treatment [4]. However, the meaning of LN in PsA is still unknown, since LN should be the place where B cells maturation process and formation of autoantibodies should take place, and PsA patients do not have specific autoantibodies.

 UA is an inflammatory condition in patients who do not fulfill classification criteria for RA, peripheral spondyloarthritis (pSpA), or PsA. Eventually, a high percentage of UA evolves into different subtypes of defined diagnostics during follow-up [8, 9]. A retrospective study showed that ST from patients with UA evolving to RA had a higher density of CD68 + macrophages and CD3 + T cells, similar to ST from established RA. On the other hand, ST from patients with UA evolving to PsA showed higher density of mast cells and lining fibroblasts [10].

In the current study, we aimed to assess the potential association between ST inflammatory cell infiltration, the presence of ectopic LN + , and poor prognostic factors (PPF) in patients with RA, PsA, and UA.

## Methods

### Patients and study design

We included patients who previously underwent knee arthroscopy or ultrasound (US)-guided biopsy. Patients were over 18 years old and fulfilled the EULAR/ACR 2010 criteria for RA or CASPAR criteria for PsA [11, 12]. We also included patients with UA that did not meet any of these criteria, after excluding infectious, crystal, or other causes of arthritis. Patients treated with biological disease-modifying antirheumatic drugs (bDMARDs) or with erosive disease were excluded. In order to standardize the longitudinal follow-up of the population included in the study, we only analyzed the clinical data from the 5 years after the synovial biopsy. We evaluated the presence of PPF during this period of time. PPF was defined in RA and UA by the presence of anti-cyclic citrullinated peptide antibodies (ACPA) and/or rheumatoid factor (RF), developed bone erosions, or need for biological therapy during the follow-up [13]. On the other hand, PPF in patients with PsA were defined as the presence of high levels of acute-phase reactants (erythrocyte sedimentation rate [ESR] and C-reactive protein [CRP]), dactylitis, or nail involvement at the time of the synovial biopsy, developed bone erosions, or need for biological therapy during the follow-up [14].

### Biopsies

Synovial biopsy specimens were obtained by needle arthroscopy selected by the presence of active synovitis of the knee or US guided of the wrist. Arthroscopy was performed with an arthroscope of diameter 2.7 mm (Storz, Tullingen, Germany). All samples were obtained from each patient from the suprapatellar pouch and the medial gutter. Biopsy specimens were fixed in 4% formaldehyde and embedded in paraffin wax. Ultrasound-guided biopsies were performed using high-sensitivity US equipment (Acuson Antares®, Siemens AG, Erlangen, Germany), using a frequency range from 10 to 12 MHz and pulse repetition frequency between 500 and 800 Hz, according to the technique described by Kelly et al. [15].

### Immunohistochemistry

Biopsies were stained with the following antibodies; T cells were labelled with rabbit antihuman CD3 polyclonal (A0452, Dako, Cambridge, UK), B cells with mouse antihuman CD20 (clone L26, DAKO), and HEV with rat antihuman PNAd (clone MECA‐79, PharMingen, Oxford, UK), anti-CD68 (macrophages, IgG1 KP1 clone; Dako), anti-CD117 (mast cells, rabbit antihuman polyclonal antibody; Dako), anti-CD15 (neutrophils, clone BY87; Novocastra), and anti-CD31 (endothelial cells, clone JC70A).

The entire area of each tissue specimen was photographed and digitized using a SPOT RT CCD camera and SPOT 4.0.4 software (Diagnostic Instruments) on an Axioplan 2 fluorescence microscope (Zeiss). For the quantitative evaluation (cells/mm^2^), tissues were sequentially photographed using Digital Image Analysis (Olympus), as previously described (10). As mentioned above, the presence of LN + is identified as radial aggregates of T and B lymphocytes with dendritic cells associated with the development of HEV with PNAd positive around a blood vessel. The highest grade of LN within each sample was determined according to a previously described scoring method [16] based on the number of radial cells count: grade 1 = 2–5 radial cells, grade 2 = 6–10 radial cells, and grade 3 ≥ 10 radial cells. LN was defined histologically as follicular aggregates grade ≥ 2 with T/B cell segregation.

### Statistical analysis

Statistical analysis was done with SPSS Statistics 27 program (Chicago, IL, USA). For the analysis of LN + and PPF, we performed *χ*^2^, *T*-test to compare PPF with the cellular density, and Spearman’s rank correlation coefficient for cellular density and disease activity score 28 (DAS28). ANOVA was used to assess the clinical and demographic characteristics among the three arthritis subgroups.

## Results

### Clinical and demographic features

A total of 88 patients were included, 26 patients with RA, 33 patients with PsA, and 29 patients with UA. A total of 59.77% were women with a mean disease duration of 60.18 (*SD* 84.2) months. Twenty-three patients (26.43%) were treated with low-dose glucocorticoids (GC), which was higher (50%) in the RA group; 40 out of 87 (45.5%) patients were treated with conventional synthetic DMARDs (csDMARDs), being methotrexate the most frequent one. The average disease activity was low to moderate in all patients, with a mean DAS28 CRP of 3.00 (*SD* 0.92) and DAS28 ESR of 3.89 (*SD* 1.19). After 5 years of follow-up, a total of 19 patients (21.6%) developed bone erosions, and 35 patients (39.8%) initiated biological therapy, especially in RA patients, while half of them required it during follow-up. Table 1 summarizes the clinical and demographic characteristics.

**Table 1:** Clinical and demographic characteristics of patients

	**Total**	**RA**	**PsA**	**UA**	***P***
*N*	88	26	33	29	
Female, *n* (%)	52 (59.77)	20 (76.92)	14 (42.42)	18 (62.06)	**0.026**
Smoking, *n* (%)	13 (14.94)	5 (19.23)	3 (9.09)	5 (17.24)	0.657
Age, mean years (SD)	50.55 (14.93)	53.2 (14.2)	51.4 (13.7)	47.24 (16.5)	0.318
Disease duration, mean months (SD)	60.18 (84.12)	83.3 (113.2)	60.7 (70.9)	29.5 (50.8)	0.581
GC, *n* (%)	23 (26.43)	13 (50)	5 (15.15)	5 (17.24)	**0.010**
MTX, *n* (%)	31 (35.22)	11 (42.30)	12 (36.36)	8 (27.58)	0.257
LEF, *n* (%)	2 (2.27)	1 (3.86)	1 (3.06)	-	0.192
SSZ, *n* (%)	4 (4.49)	-	-	4 (13.79)	**0.014**
Development of bone erosion, *n* (%)	19 (21.59)	7 (26.92)	4 (12.12)	8 (27.58)	0.181
ACPA positive, *n* (%)	27 (31.03)	19 (73.07)	-	8 (27.58)	**0.007**
RF positive, *n* (%)	24 (27.58)	16 (61.53)	-	8 (27.58)	**0.001**
HLB 27 positive, *n* (%)	15 (17.24)	-	9 (27.27)	6 (20.68)	**0.018**
DAS28 CRP mean (SD)	3.00 (0.92)	3.44 (1.1)	2.65 (0.6)	3.00 (0.9)	**0.004**
DAS28 ERS, mean (SD)	3.89 (1.19)	4.31 (1.5)	3.46 (1)	4.34 (1.2)	**0.013**
Initiation of bDMARDS, *n* (%)	35 (39.77)	13 (50)	10 (30.30)	12 (41.37)	0.301
BMI, mean (SD)	34.30 (26.27)	25 (3.84)	27.96 (3.70)	25,09 (3.33)	0.340
Dyslipidemia, *n* (%)	17 (19.31)	3 (11.53)	11 (33.33)	3 (10.34)	**0.023**
Type 2 DM	15 (17.04)	2 (7.69)	10 (30.30)	3 (10.34)	**0.019**
HBP	38 (43.18)	12 (46.15)	16 (48.48)	10 (34.48)	0.274

*GC* glucocorticoids, *MTX* methotrexate, *LEF* leflunomide, *SSZ* sulfasalazine; *ACPA* anti-citrullinated protein antibodies, *RF* rheumatoid factor, *DAS28 CRP* Disease Activity Score-28 C-reactive protein, *DAS28 ERS* Disease Activity Score-28 erythrocyte sedimentation rate, *BMI* body mass index, *T2DM* type 2 diabetes mellitus, *HBP* high blood pressure

Overall, there were no significant differences among the three groups in terms of demographic characteristics, except for the associations of seropositivity (ACPA or RF) with RA and HLA-B27 with PsA. Regarding comorbidities, PsA patients had a higher prevalence of dyslipidemia and type 2 diabetes mellitus compared to the other groups. Among the patients with PsA, five individuals (15.2%) did not display cutaneous PsO, and nail involvement was observed in five patients (15.2%). After 5 years of follow-up, 12 (41.4%), 8 (27.6%), and 4 (13.8%) patients with UA were finally diagnosed of RA, pSpA, and PsA, respectively, whereas 5 patients (17.2%) remained as UA.

### Histopathological findings

**Fig. 1:**
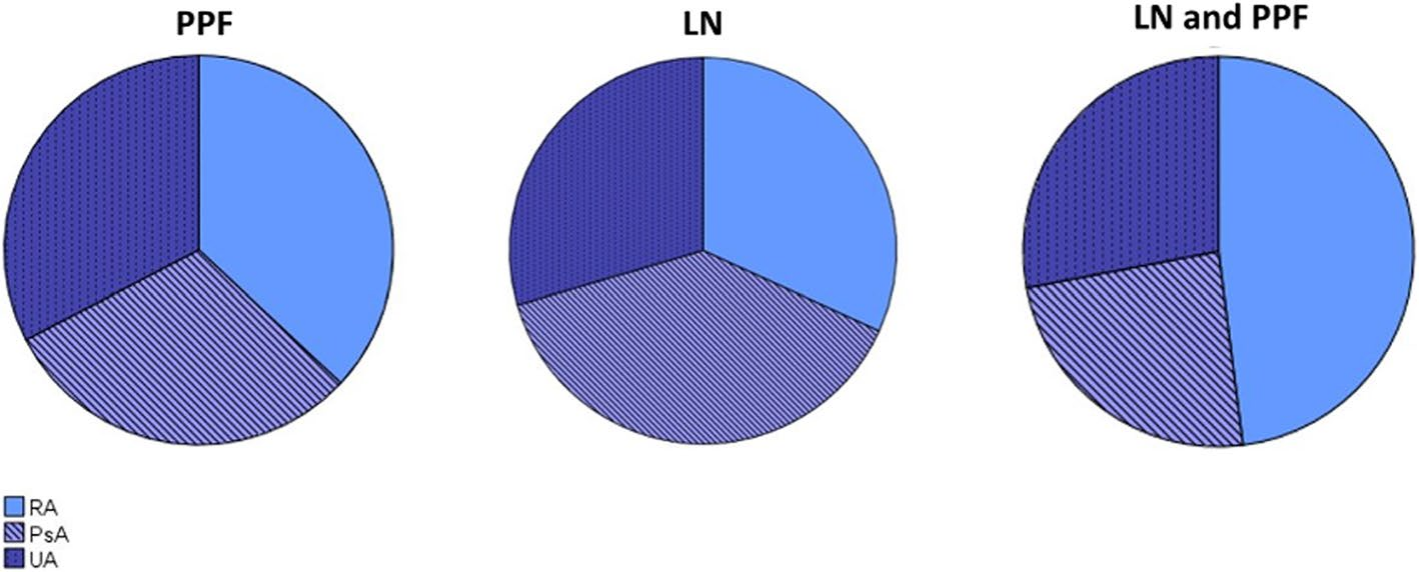
Proportion of patients with the presence of LN in the ST and PPF in RA, PsA, and UA

The majority of biopsies (84 samples, 95.5%) were obtained from the knee and 4 (4.5%) from the wrist. Overall, the distribution of PPF and the presence of LN + in the ST exhibited similarities among the three groups. Nevertheless, patients diagnosed with RA exhibited a higher prevalence of LN + , as displayed in Fig. 1.

#### Rheumatoid arthritis

Seventeen out of 26 (65.38%) RA patients had LN + , and 14 (53.84%) had PPF. Twelve patients (46%) had both LN + and PPF. LN + was associated with PPF as a group of factors (*p* 0.038). Specifically, the presence of LN + was found to have a significant association with the initiation of biologic therapy (*p* 0.018). However, no significant association was observed between the presence of LN + and the development of bone erosions (*p* 0.495) and the presence of ACPA (*p* 0.434) or RF (*p* 0.665). Furthermore, there was no association between LN + with other clinical characteristics.

There was no association between the presence of LN + and disease activity indexes, including DAS28 CRP (*p* 0.388) and DAS28 ESR (*p* 0.365). In patients with PPF, a trend of increased infiltration of inflammatory cells (CD68 + , CD3 + , CD15 + , CD31 +) was found in the ST. Nonetheless, it was not statistically significant in any of the cases, except for the elevated presence of B-cell infiltration (CD20 +) in patients needing biologic therapy (mean 320.69 [*SD* 313.14] cells/mm^2^) compared to individuals who did not require such treatment (mean 109.21 [*SD* 117.70] cells/mm^2^, *p* 0.034).

#### Psoriatic arthritis

In PsA group, we found LN + in 17 out of 33 (51.5%) and PPF in 14 patients (43.75%). Six out of 33 patients had both PPF and LN + . However, there was no statistically significant association between the presence of LN and PPF, the initiation of biological therapy, or the development of bone erosions.

**Fig. 2:**
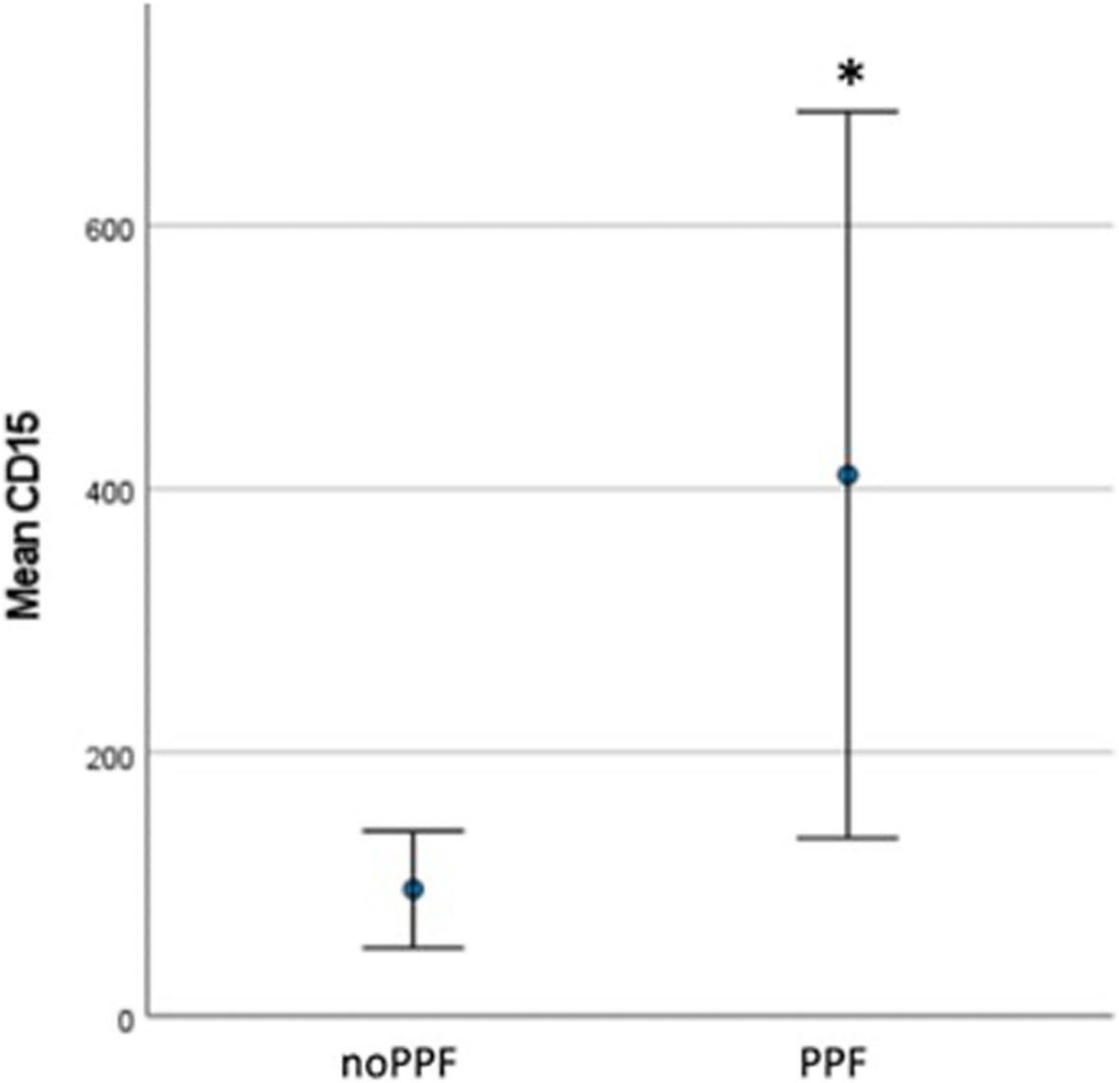
Relation between PPF and CD15 + cell in ST of PsA

Patients with PPF had higher CD15 + polymorphonuclear cell density (410.68 [*SD* 477.62] compared with patients without PPF: 96.03 [*SD* 91.95] cells/mm^2^, p 0.008) (Fig. 2). No other statistically significant associations were found. Additionally, there was no significant association between the presence of synovial cells infiltrate (CD3 + , CD20 + , CD31 + , and CD68 +) and the initiation of biological therapy or the development of bone erosions. Also, no significant correlation was identified between the number of synovial infiltrate cells (CD3, CD20 + , CD15 + , CD31 + , and CD68 +) and the DAS28, its clinical and biological components, or the duration of the disease.

#### Undifferentiated arthritis

Thirteen out of 29 (44.82%) UA patients had LN + , and 16 (55.17%) patients had PPF. Seven (24.13%) had both LN + and PPF + . LN + was not associated with PPF. There was no association between different subtypes of inflammatory cell infiltration and PPF.

ST CD68 + macrophages density was negatively correlated with DAS28 CRP (*r* =  − 0.346, *p* 0.042). Upon examining each patient individually, it was discovered that those with the highest CD68 + cell density had an average CD68 + infiltration of 1501 cells/mm^2^ (SD 443.32), whereas the remaining patients had an average of 302.93 cells/mm2 (SD 259.74). Among the individuals with the highest macrophage infiltration, the predominant final diagnosis was pSpA. These individuals exhibited only one or two swollen joints and had low or normal CRP levels (Table 2). Importantly, there were no significant differences observed between the mean CD68 + levels and various parameters, including DAS28-CRP (*p*-value 0.624), tender joint count ([TJC], *p*-value 0.405), swollen joint count ([SJC], *p*-value 0.564), or CRP (*p*-value 0.624).

**Table 2:** Clinical characteristics of patients with UA and higher CD68 + cell infiltrate

Final diagnosis	Gender	TJC	SJC	Enthesitis	CRP mg/dl	DAS28 CRP	Initiation bDMARDS	CD68 + cell/mm^2^
SpA	Female	1	1	No	0.1	2.11	Yes	1282
RA	Male	3	2	No	1.78	3.06	Yes	918
SpA	Female	2	2	Yes	0.09	2.49	No	1897
PsA	Male	1	1	No	0.01	2.08	No	1422
SpA	Male	1	1	Yes	1.04	2.36	Yes	1986

*TCJ* tender joint count, *SJC* swollen joint count, *CRP* C-reactive protein, *DAS28 CRP* Disease Activity Score-28 C-reactive protein, *bDMARDS* biological DMARDs

**Fig. 3:**
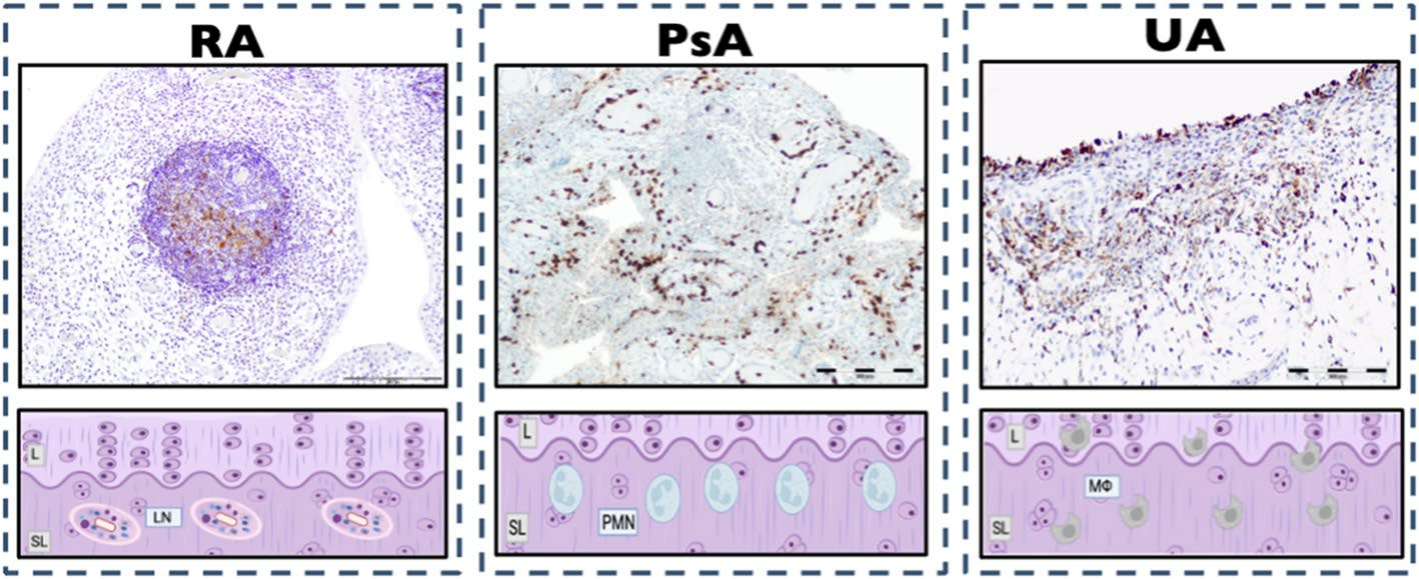
Histological findings associated with PPF in inflammatory arthritis. *PPF, poor prognostic factors; RA, rheumatoid arthritis; PsA, psoriatic arthritis; UA, undifferentiated arthritis; L, lining; SL, sublining; LN, Ectopic Lymphoid neogenesis; PMN, polymorphonuclears cells; M∅, macrophages

Figure 3 represents the histological findings associated with PPF in the three types of inflammatory arthritis.

## Discussion

The synovial membrane plays a crucial role in the immunopathology of the different subsets of inflammatory arthritis. The study of the ST has contributed to increase the understanding of their pathophysiology, distinguishing different subtypes of arthritis, identifying biomarkers, and uncovering potential therapeutic targets [10, 17–21]. Moreover, studying ST changes could help the stratification of the patients and the prediction of response/no response to targeted therapies. The study of ST is relevant as it provides unique pathophysiological information non-detectable through peripheral blood samples. While the presence of ACPA or RF antibodies has traditionally been considered a poor prognostic factor in RA, it is important to note that not all RA patients exhibit these antibodies, and they may still develop erosive disease. On the other hand, in PsA and UA, there is a lack of specific blood biomarkers indicative of poor prognosis or response to therapy. Therefore, the study of ST becomes relevant.

Although previous studies have documented the characteristics of ST in inflammatory arthritis, our research focuses on providing a comparative analysis of ST characteristics in different subsets of inflammatory arthritis and exploring their correlation with PPF. Consistent with prior research, the presence of LN + is specifically linked to PPF in patients diagnosed with RA, particularly in relation to the initiation of biological therapy [2, 3]. These findings indicate the distinct pathogenic role that these lymphoid aggregates may have in RA. Accordingly, previous studies have shown that the increase in the expression of lymphoid-associated genes in the ST is correlated with autoantibody positivity, while elevated levels of osteoclast-targeting genes predict the progression of radiographic joint damage [22]. In our study, we did not observe any association between the presence of LN + and autoantibodies or the development of bone erosions, probably due to the limited number of patients with bone erosions.

Our study revealed a correlation between the presence of PPF and the density of CD15 + polymorphonuclear cells in the ST of patients with PsA, highlighting the significant role of myeloid cells in the pathogenesis of PsA. Treatment response in SpA patients is associated with reduction in IL-17 expressing mast cells [23], while both SpA and PsA patients commonly exhibit IL-17A + neutrophils (CD15 + cells) [24]. Although LN + is associated with PsA and treatment response in previous studies [4], our current study did not find a significant association between LN and PPF.

Compared to patients with RA, the synovial membrane of patients with PsA exhibits distinct characteristics. Specifically, the lining of the synovium in PsA patients has fewer cell layers, primarily composed of synovial fibroblasts and CD68 + macrophages [25]. On the other hand, the sublining of PsA patients is similar to RA, showing a higher density of CD163 + macrophages, increased vascularity, and a greater abundance of neutrophils and mast cells [26, 27].

In contrast, in our cohort of patients with UA, the presence of CD68 + macrophages in the ST showed a negative correlation with DAS28-CRP, indicating lower systemic activity. To further investigate these findings, our analysis revealed that patients exhibiting a higher CD68 + infiltrate were those diagnosed with pSpA, characterized in our cohort by the presence of only one inflamed joint and normal CRP levels. This suggests a localized inflammatory activity within the ST with limited systemic burden. Consequently, this could be a possible explanation for their activity score which indicated either remission or low disease activity, despite having high local activity in the joint. These findings align with previous studies demonstrating a correlation between CD68 + cell infiltrates and ultrasound-assessed inflammatory activity [28].

The results of this study should be carefully interpreted since there are some important limitations. The retrospective design and the relatively small cohort of patients are the main hurdles to draw strong conclusions. Additionally, some patients had underwent systemic treatment with csDMARDs, which may have influenced the findings in the ST. Nevertheless, this study provides a comprehensive assessment of inflammatory arthritis, examining the common pathogenic mechanisms shared by RA, PsA, and UA while considering their distinct PPF. Although this study has a retrospective design, the data collection was robust with rigorous exclusion criteria, including patients with active arthritis, ensuring access to a substantial amount of clinical, demographic, and immunohistochemical data from ST. Additionally, the follow-up period was extended up to 5 years after the biopsy, offering valuable insights into the long-term progression of these diseases.

With the arrival of new biotechnologies, ST studies have been expanded beyond immunohistochemistry. Therefore, our results could differ if using new techniques for synovial analysis. Recently, a stratified, biopsy-driven, multicenter, open-label, phase 4 randomized controlled trial found that patients with few or no B-cell gene expression in the ST had higher response rates to tocilizumab compared to rituximab therapy [29]. A further synovial biopsy-based biomarker analysis of the same study reported ST genetic signatures associated with response to rituximab, response to tocilizumab, response to both therapies, or non-responding at all (refractory disease). On the other hand, the authors reported a synovial fibroblast population associated to the pauci-imume synovial phenotype and to refractory disease, which could be of interest as biomarker and therapeutic target [30].

Recent transcriptomic studies employing single-cell RNA sequencing have illuminated disparities in synovial tissue of individuals with active RA and PsA [31]. However, progress in ST research and standardization of its practical application remains a challenge. Nonetheless, immunohistochemistry has become more widely accessible, making its utilization in current clinical practice more feasible. However, we emphasize the indispensable role of omics techniques in deepening our understanding of ST in inflammatory arthritis, consequently advancing in personalized medicine. Yet, clinical trials based on molecular pathology in PsA and UA remain a notable gap, with their incorporation considered crucial for future investigations. Ultimately, the findings from ST studies may guide treatment decisions in routine clinical practice.

## Conclusion

This study highlights the potential utility of ST analysis in predicting the prognosis of patients with inflammatory arthritis. LN + and specific cell types in the synovium may serve as valuable indicators of disease progression and the need for advanced therapeutic interventions. However, further research is needed to validate these findings and their clinical implications.

## Data Availability

The data analyzed during the current study are available from the corresponding author (JR) upon reasonable request.

## Abbreviations

RA: Rheumatoid arthritis
PsA: Psoriatic arthritis
UA: Undifferentiated arthritis
ST: Synovial tissue
LN +: Ectopic lymphoid neogenesis
PNAd +: Peripheral node Addressin positive
HEV: High endothelial venules
TNFi: TNF inhibitors
pSpA: Peripheral spondyloarthritis
bDMARDs: Biological Disease-modifying antirheumatic drugs
PPF: Poor prognostic factor
ACPA: Anti-cyclic citrullinated peptide antibodies
RF: Rheumatoid factor
ESR: Erythrocyte sedimentation rate
CRP: C-reactive protein
US: Ultrasound
DAS28: Disease activity score of 28 joints
GC: Glucocorticoids
csDMARDs: Conventional synthetic DMARDs
